# Scenarios for Assessing Leadership Behaviors of Mid-level Managers in the Greek National Health System Through the Lens of Servant Leadership Theory: A Pilot Study in the Hospital Cluster of North Attica

**DOI:** 10.7759/cureus.60438

**Published:** 2024-05-16

**Authors:** Emmanouil Koutalas, Evangelos Kostares, Eva Paraskevadaki, Kyriakos Souliotis, Vasiliki Koumaki, Sophia Kalantaridou, Athanasios Tsakris, Maria Kantzanou

**Affiliations:** 1 Department of Microbiology, National and Kapodistrian University of Athens School of Medicine, Athens, GRC; 2 Department of Research, Health Policy Institute, Athens, GRC; 3 Department of Social and Education Policy, University of Peloponnese, Corinth, GRC; 4 Third Department of Obstetrics and Gynecology, Attikon Hospital, National and Kapodistrian University of Athens School of Medicine, Athens, GRC

**Keywords:** stewardship, healthcare, greece, behavior, managers, servant leadership theory

## Abstract

This study aimed to develop a scenario-based questionnaire for evaluating medium-level leadership behaviors within the Greek National Healthcare System (NHS), drawing upon the principles of servant leadership theory. Data for this pilot study were collected in the first quarter of 2019, using a sample of 33 (22.9% of all medium-level managerial positions) medium-level managers from the Greek NHS hospital cluster located in North Attica. To assess managerial behaviors, an ordinal scale was employed, revealing non-normal data distributions. Consequently, our analysis involved presenting descriptive statistics, utilizing non-parametric tests to explore distinctions in managerial behaviors, and conducting thematic analysis of responses to open-ended questions, with frequencies and relative frequencies of each theme meticulously recorded. Overall, our findings indicate that, in most cases, managers exhibited positive behaviors toward their employees, regardless of whether the outcomes were positive, negative, or unknown. Positive behaviors towards the administration were comparatively rare. Significant differences were observed, highlighting that managers were more inclined to exhibit positive behaviors when the outcome was known, particularly in scenarios involving employee management. Within each scenario, behavioral patterns varied, with managers demonstrating a propensity to take credit for employee success in positive outcomes but distancing themselves from negative outcomes when reporting to the administration. Furthermore, the survey responses underscored the prevalence of positive attitudes regarding accountability and stewardship, with stewardship showing a positive correlation with scenario-based behaviors. Finally, our study brought to light several challenges in the management of the Greek NHS, including the absence of comprehensive managerial evaluation, the lack of meritocracy, regulatory deficiencies, and a shortage of leadership skills among current managers. These findings emphasize the importance of scenario-based assessments for Greek hospital managers, as they can help connect managerial behaviors to stewardship, accountability, and skills, ultimately contributing to the enhancement of leadership within the Greek NHS.

## Introduction

The Greek National Healthcare System (ESY, Greek NHS) has faced prolonged challenges in resource management and corporate governance, leading to sustainability concerns [1]. These issues have been exacerbated by the aftermath of the 2008 economic crisis and the recent strain imposed by the refugee crisis [2]. The healthcare workforce in Greece has also borne the brunt of the economic crisis, impacting working conditions for healthcare professionals [3]. Consequently, Greek public hospitals have struggled to meet patient needs even before the onset of the COVID-19 pandemic, creating a substantial gap between patient expectations and actual experiences [3,4].

Effective leadership plays a critical role in healthcare settings, influencing the coordination, integration, and quality of patient care [5]. However, within the Greek NHS, leadership shortcomings have been reported. Managerial appointments are often influenced by political considerations rather than essential skills and experience, resulting in outdated leadership behaviors and practices, which contribute to an adverse organizational culture and reduced motivation. Recent research has demonstrated that these issues hinder ongoing learning [6], a crucial component of professional growth, particularly in healthcare settings where teamwork and employee relationships significantly impact performance [7,8].

The existing body of research on organizational leadership has primarily focused on elements and methodologies contributing to effective leadership within organizational environments [9,10]. Ineffective leadership, often referred to as the "dark side of leadership," is associated with characteristics such as narcissism, self-centered behaviors, improper use of authority, and one-way communication [11]. However, our understanding of what renders leaders ineffective and the specific ways in which they impact organizational behaviors and outcomes remains limited. Ineffective leadership stands in contrast to organizational interests, leading to reduced performance and efficiency, negatively affecting employees' psychological well-being, commitment, self-assurance, motivation, and job satisfaction. This phenomenon, often characterized as the "toxic triangle," results from the interplay between unfavorable employee traits, ineffective leadership, and problematic organizational contexts [12]. In the context of Greek hospitals, additional components of the toxic triangle may include officers functioning as compliant followers with unmet needs for incentives and a toxic environment characterized by limited mobility, inadequate corporate governance structures, and human resource policies [12].

Transformational leadership has been identified as the most effective style for healthcare services [13]. To assess leadership styles, tools like the Multifactor Leadership Questionnaire (MLQ-6S), the Authentic Leadership Questionnaire (ALQ), and the Servant Leadership Scale (SLS) are employed. These instruments help in evaluating self-awareness, transparency, ethics, and leader-follower relationships in the healthcare context [14-16]. Additionally, clinical leadership has been introduced for staff nurses in healthcare settings, emphasizing the importance of relationship- and communication-oriented leadership [17].

This paper focuses on medium-level managers' behaviors in the leader-follower relationship, particularly in terms of ethical and people-centered practices of leaders within the Greek NHS. The servant-leadership theory encompasses eight key dimensions of leadership, including empowerment, accountability, standing back, humility, authenticity, courage, interpersonal acceptance, and stewardship. While previous research has predominantly relied on the perceptions of managers and employees to assess leadership practices, this study proposes using scenario-based evaluation to provide a more accurate representation of actual behaviors and potential organizational shortcomings.

Despite the acknowledged importance of leadership in organizational performance, there is a dearth of evidence regarding leadership behaviors and practices within the Greek NHS, despite the complex challenges posed by the hospital workplace context. This pilot study aims to develop scenarios for evaluating leadership behaviors of medium-level managers within the framework of servant-leadership theory. Furthermore, it examines how these scenario behaviors relate to attitudes and perceptions of accountability, stewardship, and managerial competencies, providing insights into the relationship between real-life behaviors and perceived leadership practices in the Greek hospital environment, which is known to present strong leadership neutralizers.

## Materials and methods

Sample

In the first quarter of 2019, a cross-sectional multicenter study was conducted in Greece. The procedure of data collection was authorized by the three Scientific Committees of the Hospital Units. All managers received via email the link to the electronic version of the questionnaire, along with a detailed description of the objective of this study and information regarding the voluntary nature and anonymity of participation and the secured handling of collected data.

The sample of this pilot study consisted of 33 medium-level managers of the Greek NHS hospital cluster in North Attica (hospital units of Sismanoglio, Amalia Fleming, Paidon Pentelis), which accounts for 22.9% (33/144) of medium-level managers’ positions in that specific hospital cluster. To facilitate participation and ensure the authenticity of the answers, demographic data (gender, age, education, etc.) were not gathered by the questionnaire in this study.

Development of data collection instrument

In light of the considerable challenges posed by the absence or dysfunction of business plans and objectives in Greek NHS hospitals, evaluating leadership performance becomes a complex task. However, the primary objective of this pilot study was to develop scenarios aimed at addressing the leader-follower relationship, focusing specifically on ineffective leadership traits such as self-serving and self-centered behaviors and one-way communication [11]. Given the intricate and pivotal role of leader-follower relationships in the complex hospital environment, where performance and organizational objectives are at stake, scenario-based assessments of managers' behaviors toward employees (lower-level) and administration (higher-level) may provide a more insightful understanding of the true characteristics of medium-level managers' leadership. At the heart of effective leadership lie principles of accountability and stewardship [18]. Scenarios crafted around these principles are intended to shed light on medium-level managers' perspectives regarding the instability and level of threat within the Greek NHS organizational context. To achieve this objective, the questionnaire was divided into several sections.

The first part of the questionnaire comprised four scenarios, as detailed in Table 1. The first two scenarios were constructed based on hypothetical initiatives by subordinates, with known outcomes (positive or negative), and respondents reported their corresponding behavior toward both administration and employees. In the third scenario, the outcome of the subordinate's hypothetical initiative remained unknown. The fourth scenario introduced a hypothetical institutional change in the appointment and evaluation of managers, and respondents were asked to express their level of support. The responses were rated on a scale of 0 (reflecting negative behavior) to 2 (indicating positive behavior), with the aim of assessing the extent to which servant leadership and inefficient leadership traits were exhibited [10,11].

**Table 1:** Scenarios Developed for Evaluating Managerial Behaviors in Terms of Servant Leadership Theory

Scenario	Response Option	Rating	Justification
A: Your subordinate, in a position of responsibility, takes an initiative within the limits of his/her authority without informing you in time for approval, leading to a positive result.			
A1) Subordinate takes initiative leading to positive results without prior approval.	a) Not mentioned at all.	0	Passive self-serving behavior towards both supervisor and subordinate related to passive/avoidant leadership, lack of courage, and ineffective leadership.
	b) Mentioned to a small extent.	1	Neutral behavior indicating low levels of authenticity, i.e., representing inaccurately the true situation.
	c) Mentioned in some detail.	2	Positive behavior reflects self-confidence, humility, standing back, and high levels of authenticity and stewardship.
	d) Mentioned as a fully independent initiative.	1	Neutral behavior reflects some level of authenticity but lower levels of courage and stewardship which may be indicative of a passive/avoidant leadership style.
	e) Mentioned as a fully independent initiative.		
A2) Reaction to being informed about positive outcome.	a) Reprimand for not informing in advance.	0	Negative behavior with low levels of stewardship, interpersonal acceptance, courage, and empowerment. Inability to build a cohesive team, being overtly emotional, and maintaining poor relations with staff.
	b) Reward for the positive outcome.	1	Neutral behavior, high levels of interpersonal acceptance and empowerment yet limited stewardship.
	c) Both of the above.	2	Positive behavior indicating high levels of stewardship and accountability.
B: The initiative leads to failure with negative consequences, and the subordinate informs you afterward.			
B1) Initiative leads to failure with negative consequences.	a) Not mentioned at all.	0	Negative self-serving behavior towards both supervisor and subordinate related to lack of stewardship, courage, ineffective leadership, and undermanaging.
	b) Mentioned to a small extent.	2	Positive behavior showing accountability, courage, authenticity, and stewardship.
	c) Mentioned quite extensively.	1	Neutral behavior showing some level of authenticity but low interpersonal acceptance and limited stewardship.
	d) Mentioned as a fully independent initiative.	0	Negative behavior as it reflects a lack of stewardship and interpersonal acceptance, i.e., a self-serving behavior related to ineffective leadership
B2) Reaction to being informed about the initiative's negative outcome.	a) Reprimand for not informing in time, and prevent further initiatives.	2	Positive behavior with high levels of accountability, authenticity, and stewardship.
	b) Report for further disciplinary action.	1	Neutral behavior since the manager shows some level of authenticity but no accountability, as well as indifference to interpersonal acceptance.
	c) Both of the above.	0	Negative behavior since the manager exhibits wrongful use of power, intimidation, and coercion while showing low levels of accountability, interpersonal acceptance, and courage.
C: Subordinate takes an initiative marginally within the scope of authority, assuming disagreement due to risk, and reports it in progress (Initiative taken with knowledge of risk and without prior approval).			
	Covering and supervising actions to minimize risks.	2	Positive behavior indicating high levels of empowerment, accountability, courage, interpersonal acceptance, stewardship, and standing back.
	Report it directly to management.	0	Negative behavior as it reflects low accountability, courage, interpersonal acceptance, and stewardship.
	Instruct to stop it and comply.	1	Neutral behavior since the manager shows some level of stewardship yet less empowerment, courage, and interpersonal acceptance.
	Cover actions and report it.	2	Positive behavior indicating high levels of empowerment, accountability, courage, interpersonal acceptance, and stewardship.
	Report and instruct to stop.	0	Negative behavior as it reflects low accountability, courage, interpersonal acceptance, as well as intimidation (ineffective leadership traits).
D: Hypothetical institutional change in the managerial position framework (increased powers, salary, contract terms, evaluation) (support for institutional change in managerial position).			
	Reject.	0	Negative behavior indicating a limited level of accountability, stewardship, courage, humility, and standing back.
	Neither support nor reject.	1	Neutral behavior indicating some level of accountability, stewardship, courage, humility, and standing back.
	Support.	2	Positive behavior indicating a high level of accountability, stewardship, courage, humility, and standing back.

The second part of the questionnaire delved into managers' attitudes concerning accountability and stewardship, key dimensions of the servant leadership theory, which emphasizes the moral aspects of leadership. Given the absence of a performance appraisal process in the context of the Greek NHS that assesses leaders' moral qualities and values, two questions were posed: "To what extent do you cover any mistakes of your subordinates to your supervisor by ultimately accepting responsibility" and "To what extent do you consider the failures and/or mistakes of the team you supervise to be your own mistakes and failures?" These questions were rated on a five-point Likert scale (1 = never, 5 = always) (Table 2).

**Table 2:** Investigating Accountability and Stewardship

Question	Rating Scale (1=Not at All, 4=Quite a Lot)
To what extent do you cover any mistakes of your subordinates to your supervisor by ultimately accepting responsibility?	
To what extent do you consider the failures and/or mistakes of the team you supervise to be your own mistakes and failures?	

The third part of the questionnaire probed managers' perceptions regarding the skills and competencies associated with effective team management (Table 3). Participants were asked to rate, on a 4-point Likert scale (1 = not at all, 4 = quite a lot), the extent to which they delegate and decentralize tasks, authorize subordinates to take initiatives, supervise and coach in the execution of relevant actions, provide feedback, and reward employees when they succeed. Effective managers must possess the requisite knowledge, skills, and abilities to execute various managerial tasks [19]. In hospital settings, mid-level managers formulate functional-level strategies, communicate them to subordinates, translate tasks into actions, and supervise employees in their execution [20]. This process is underpinned by a culture of accountability that permeates all levels of the hierarchy. In the healthcare context, stewardship, accountability, and responsibility to both the organization and patients are essential for the effective management of human and financial resources [21]. Delegation is a critical leadership quality, and it is vital for managers to delegate effectively [22,23]. A clear and consistent performance framework, encompassing training, coaching, planning, bi-directional feedback, reviews [24], motivation, recognition, and rewards [23,25], should be followed to achieve high performance.

**Table 3:** Evaluating Managerial Skills and Competencies for Effective Team Management

Skill/Competency	Rating Scale (1=Not at All, 4=Quite a Lot)
Delegating and decentralizing tasks	
Extent of task delegation and decentralization	

In the final section of the questionnaire, an open-ended question provided participants with the opportunity to identify and discuss the three most significant deficiencies in the current model of Administration and Operation of Public Hospitals and offer suggestions for enhancing Corporate Governance (Table 4).

**Table 4:** Participant Suggestions for Enhancing Corporate Governance in Public Hospital Administration and Operation

Question	Reply Section
Identify the three most significant deficiencies in the current model of administration and operation of public hospitals.	
Discuss why these deficiencies are significant.	
Offer suggestions for enhancing Corporate Governance in public hospitals.	

Ethics statement

The study received approval from the Ethics Committees of the Greek NHS hospital cluster in North Attica, encompassing the hospital units of Sismanoglio, Amalia Fleming, and Paidon Pentelis, under EC number 22520 on October 30, 2018. The research was conducted following the principles of the 1964 Declaration of Helsinki and its later amendments.

Statistical analysis

The items of the managerial behavior scenarios were scored on an ordinal scale (0 = negative, 1 = neutral, 2 = positive), and the total score for managerial behaviors was calculated as an unweighted sum. The Shapiro-Wilk test was used to examine the normality of the distributions for managerial behavior and perceptions and attitudes variables, showing that the distribution could not be considered normal (p < 0.05). Hence, descriptive statistics in terms of absolute and relative frequencies (N, %), as well as median and IQR values, were reported, and non-parametric tests were utilized in the analyses. More specifically, the paired samples Wilcoxon test was used to detect possible differences in managerial behaviors between the three outcome scenarios, and Spearman’s rho coefficient was calculated for the correlations between leadership perception variables. The responses to the open-ended questions were analyzed thematically, and the frequencies and relative frequencies of each theme were reported.

## Results

Scenarios

Frequencies (N) and relative frequencies (%) of managerial behaviors in different scenarios are presented in Table 5. A proportion of 84.8% and 78.8% of managers adopted positive behaviors toward employees for initiatives with positive and negative outcomes, respectively. On the other hand, only 3.0% (positive outcome) and 27.3% (negative outcome) of managers adopted positive behaviors toward administration. For initiatives with unknown outcomes, only 27.3% of managers presented positive behaviors, while for the scenario of promotion and meritocracy, 57.6% reported positive behaviors.

**Table 5:** Frequencies and Relative Frequencies of Managerial Behaviors at Different Scenarios

Scenario	Negative		Neither Negative nor Positive		Positive	
	N	%	N	%	N	%
A1. Initiative with positive outcome (Report to Administration)	1	3.00%	31	93.9%	1	3.00%
A2. Initiative with positive outcome (Employee Management)	0	0.00%	5	15.20%	28	84.80%
B1. Initiative with negative outcome (Report to Administration)	8	24.20%	16	48.50%	9	27.30%
B2. Initiative with negative outcome (Employee Management)	6	18.20%	1	3.00%	26	78.80%
C. Initiative with unknown outcome (Employee Management)	21	63.6%	3	9.10%	9	27.30%
D. Manager’s performance appraisal	5	15.20%	9	27.30%	19	57.60%

Bivariate comparisons between scenario scores are presented in Table 6. Results showed a significant difference (Z = 4.463, p < 0.001, r = 0.788) between employee management behaviors for positive and unknown outcomes, where managers adopted more positive behaviors when the outcome of the employee initiative was assumed to be positive. The same pattern was evident even if the outcome of the initiative turned out negative (Z = 3.461, p = 0.001, r = 0.611), indicating that overall, a possible unknown outcome is linked to more negative managerial behaviors toward the employees, compared to a known outcome, either positive or negative. Moreover, the effect sizes of the differences between known and unknown outcomes are large, meaning that the level of uncertainty of the outcome could cause a large shift in managerial behavior.

**Table 6:** Median Values and Interquartile Ranges for Employee Management and Report to Administration Behaviors at Different Scenarios NA: not applicable

Scenarios	Employee Management	Report to Administration	Z	p
A. Employee’s Initiative: Positive outcome	2.00(0.00)	1.00(0.00)	-4.952	<0.001
B. Employee’s Initiative: Negative outcome	2.00(0.00)	1.00(1.50)	-2.899	0.004
C. Employee’s Initiative: Unknown outcome	0.00(2.00)	0.00(2.00)	-4.463	0.000
D. Manager’s performance appraisal	2.00(1.00)	2.00(1.00)	-2.562	0.010
Comparison: Positive vs. Negative Initiative	NA	NA	-1.838	0.066
Comparison: Positive vs. Unknown Initiative	NA	NA	-3.461	0.001
Comparison: Negative vs. Unknown Initiative	NA	NA	-1.914	0.056
Comparison: Positive Initiative vs. Manager	NA	NA	-2.595	0.009
Comparison: Negative Initiative vs. Manager	NA	NA	-2.746	0.006
Comparison: Unknown Initiative vs. Manager	NA	NA	-3.284	0.001

Significant differences were also reported within each outcome scenario, between behaviors adopted in employee management and reporting to administration. For the positive outcome scenario, more negative behaviors were adopted in reporting to administration (Z = 4.952, p < 0.001, r = 0.875), meaning that managers tend to acquire part of employees’ success when convenient. Also, in the negative outcome scenario, managers tend to disclaim the negative effects of their employees’ initiative by fully reporting their subordinate’s actions, leading to more negative behavior in reporting to the administration (Z = 2.899, p = 0.004, r = 0.504), compared to employee management behaviors which are more positive in general. In the promotion and meritocracy scenario, behaviors were more negative compared to employee management in the positive outcome (Z = 2.562, p = 0.010, r = 0.446). On the other hand, behaviors in the promotion and meritocracy scenario were more positive compared to reporting to administration in the positive outcome (Z = 2.746, p = 0.006, r = 0.485), reporting to administration in the negative outcome (Z = 2.595, p = 0.009, r = 0.458), and the scenario of unknown outcome (Z = 3.284, p = 0.001, r = 0.580), indicating that managers would support contexts where promotion is based on results, yet they adopt different behaviors in the present context toward administration (in either positive or negative outcomes) and in employee management when the outcome is unknown.

Attitudes and perceptions for managerial skills/competencies

On average, positive attitudes were reported for attitudes toward accountability (median = 4, interquartile range (IQR) = 1) and stewardship (median = 4, IQR = 2), as well as perceptions of the following skills/competencies: reward (median = 4, IQR = 1), feedback (median = 3, IQR = 1), coaching (median = 3, IQR = 1), assignment (median = 3, IQR = 1), and delegation (median = 3, IQR = 1), as presented in Table 7. Nevertheless, Spearman correlations (Table 8) indicated that only attitude toward stewardship was linked to more positive scenario behaviors (r = 0.574, p < 0.05).

**Table 7:** Mean and Median Values for Managers’ Attitudes and Perceptions IQR: interquartile range

Attitudes	Min	Max	Mean	SD	Median	IQR
Accountability	1	5	3.70	0.88	4.00	1.00
Stewardship	1	5	3.58	0.94	4.00	2.00
Perceptions						
Delegation	1	4	3.30	0.47	3.00	1.00
Assignment	1	4	3.30	0.65	3.00	1.00
Coaching/mentoring	1	4	3.33	0.48	3.00	1.00
Feedback	1	4	3.21	0.65	3.00	1.00
Reward	1	4	3.64	0.49	4.00	1.00

**Table 8:** Spearman Correlation Coefficient Results Between Leadership Perceptions Items * p< 0.05, ** p< 0.01.

	1	2	3	4	5	6	7	8
Scenario scores	-							
Accountability	-.007	-						
Stewardship	0.554**	0.116	-					
Delegation	-0.146	-0.026	0.079	-				
Assignment	-0.156	0.150	0.118	0.561**	-			
Coaching/mentoring	-0.275	-0.014	-0.286	-0.047	0.020	-		
Feedback	-0.123	-0.361*	0.002	0.066	-0.051	0.476**	-	
Reward	-0.109	-0.039	0.174	-0.05	0.106	0.000	0.152	-

Challenges for Greek NHS

**Figure 1:**
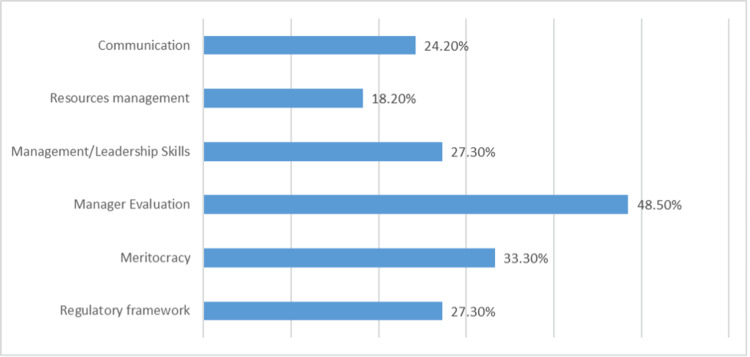
Challenges Reported by Managers for the Greek NHS NHS: National Healthcare System

Finally, managers were asked to report, through an open-ended question, the challenges of the current situation in Greek NHS management. Their responses were grouped according to thematic content, and the results are presented in Figure 1. The lack of managers’ evaluation was reported by 48.5% of participants, followed by a lack of meritocracy (33.3%), shortcomings and overlapping in the regulatory framework (27.3%), and limited leadership skills of current managers (27.3%).

## Discussion

This study aimed to propose a scenario-based questionnaire to assess medium-level leadership behaviors in the Greek NHS, based on the servant leadership theory. Additionally, scenario outcomes were associated with attitudes and perceptions reported by leaders to showcase possible differences between managers’ perceptions, attitudes, and behaviors in simulated real-life scenarios. The behaviors of medium-level managers reported in scenarios of negative and positive outcomes of employee initiatives were better toward employees compared to administration. More specifically, managers expressed higher levels of stewardship, authenticity, and accountability in their relationship with their subordinates, while demonstrating lower levels of courage and authenticity, as well as self-serving behaviors, toward the administration.

In the Greek NHS, medium-level managers and employees have very limited job mobility, resulting in colleagues working together for many years and developing a sense of support. However, upper-level managers have a very high fluctuation according to the change of political leadership, including the CEO and the administrative board of the hospital [26]. Therefore, there is a lack of administrative continuity in the hierarchy of the Greek NHS, creating a threatening organizational context for mid-level managers. This leads to opportunistic and self-serving behaviors to defend and maintain their positions, regardless of the fluctuations of the upper management due to governmental changes [11].

Similarly, when the simulated outcome of the employee’s initiative was unknown, medium-level managers exhibited more self-serving behaviors, such as lower levels of courage, interpersonal acceptance, and stewardship in their relationship with their subordinates, compared to their behaviors reported for known outcomes (either positive or negative). It is indicated, therefore, that the unknown, in general, fortifies the level of perceived threat from the organizational environment, acting as a strong neutralizer for effective leadership [12]. Moreover, managers acknowledge the lack of a meritocracy framework in the Greek NHS, so their behavior is influenced by their willingness to protect their positions from administrative political changes and the inconsistency of political priorities according to governmental changes. Although they know the basics of managing subordinates, as showcased by their behaviors in known outcome scenarios, increased levels of threat in the organizational context lead to negative defensive behaviors.

No significant relationship was observed between scenario behaviors reported by medium-level managers and their attitudes toward accountability or their perceptions regarding skills and competencies. This shows that a positive attitude toward accountability and high levels of perceived managerial skills and competencies do not necessarily relate to actual behaviors in a real-life scenario. On the one hand, this result highlights the importance of assessing leadership behavior with quasi-experimental methods, and on the other, it leads to a discussion about the organizational, contextual factors that affect these behaviors. For example, a recent study of the Greek NHS, including six public Greek hospitals, indicated that the organizational culture and management practices do not support continuous learning and self-development, while performance is poor and the leadership weak and ineffective. This study concluded that different and consistent leadership practices and human resource policies should be adopted horizontally and vertically across all functions to improve organizational learning, teamwork, accountability, and higher performance [4].

Yet, a significant correlation was reported between scenario behaviors and the extent to which managers consider the failures and/or mistakes of their team to be their own mistakes and failures. This indicates that stewardship, as a core element of leadership, may lead to more positive behaviors toward subordinates and the administration of the hospital. According to the World Health Organization (WHO), stewardship as a leadership behavior is the most effective and efficient approach to strengthening healthcare systems because it improves responsibility, assures equity, and better serves society's needs in terms of public healthcare [18].

In the complex and multidisciplinary hospital environment, particularly within the Greek NHS, there are multiple typical and atypical centers of power and different sets of interests without a consistent corporate plan and aligned vision across all hospital units and departments. Therefore, it is worth questioning whether the Greek NHS indeed has ineffective medium-level managers or if the negative behaviors reported in this study can be attributed to strong contextual neutralizers of effective leadership. This question becomes more prominent when considering the shortcomings reported by managers themselves, such as the lack of evaluation and meritocracy, as well as the limited leadership skills of current managers. For example, in their study of public sector organizations in Greece, researchers assert that leadership quality is not necessarily the problem in public organizations [27]. Instead, factors such as bureaucratic controls, external political influence, and limited positive reward power are the factors that neutralize the effectiveness of leadership [28,29]. Yet, some elements of servant leadership may exist in the Greek NHS due to the Public Service Motivation level [30], which includes the qualitative elements that motivate employees to go beyond self-interest to serve the public. In this frame, effective leadership may address the sense of public duty in public hospital employees, which in turn could support and optimize the levels of servant leadership in the Greek NHS.

Limitations

This study is one of the first in Greece to propose scenario-based evaluation of managers' behavior in the Greek NHS, based on the servant leadership theory that addresses the ethical and people-centered practices of leaders. However, this study presents some limitations. First, even though the sample was more than 20% of the target population, it remains small (N=33), limiting the extent to which the reliability of the instrument could be assessed. Due to the limited target population, demographic characteristics of the sample were not requested to avoid implications related to the anonymity of participants. Additionally, the sample of this study was limited to managers of a single hospital cluster in Greece, and due to the nature of the sampling procedure, sampling bias may be an issue since only those managers who were willing to participate in the study completed the questionnaires. It should also be mentioned that the scenarios proposed in this study address issues of servant leadership theory by focusing on the relationships between medium-level managers, the administration, and subordinates, leaving room for additional scenarios to further investigate.

## Conclusions

In this pilot study, we conclusively highlight the necessity for the Greek NHS to undergo a significant leadership overhaul to ensure sustained high performance. The cornerstone of this transformation is a top-down initiated paradigm shift, focusing on reforming the selection and appointment process for hospital top managers. Emphasizing the importance of skills, competencies, and a proven performance track record is critical. The study advocates for rigorous performance evaluations of managers to ensure their strategies are aligned with the overarching business goals of hospitals.

The evidence presented underscores the effectiveness of scenario-based evaluations in assessing the behaviors of mid-level managers within the Greek NHS, spotlighting the pivotal roles of stewardship and accountability. It calls for a meritocratic approach to managerial appointments, suggesting that aligning managerial actions with stewardship values paves the way for enhancing healthcare system performance. Implementing these scenario-based evaluations for developing managerial skills and competencies could serve as a strategic tool in addressing the current challenges impeding effective leadership within the Greek NHS.
